# Data on species and concentration of the main gaseous products during sludge combustion to support the feasibility of using sludge as a flue gas denitration agent for the cement industry

**DOI:** 10.1016/j.dib.2019.103998

**Published:** 2019-05-24

**Authors:** Zijun Tang, Ping Fang, Xiang Xiao, Jianhang Huang, Xiongbo Chen, Peiyi Zhong, Zhixiong Tang, Chaoping Cen

**Affiliations:** a South China Institute of Environmental Sciences, Ministry of Ecology and Environment, Guangzhou 510655, China; b The Key Laboratory of Water and Air Pollution Control of Guangdong Province, Guangzhou 510655, China

**Keywords:** Sludge, Gaseous products, Combustion, Cement industry

## Abstract

The dataset presented in this article is the supplementary data for the research article Fang et al., 2019 [1] and provided detailed data profile to support that sludge is an effective NO_X_ reducing agent, as reductive gas components produce during sludge combustion. The instantaneous concentrations of the main gaseous products during sludge combustion were detected by using Fourier transform infrared spectroscopy (FTIR, DX-4000, Gasmet Technologies). The results showed the distribution and concentration level of gaseous products during sludge combustion and evidenced the feasibility of using sludge as a deNO_X_ agent in cement industry.

**Table:** Specifications table

Subject area	*Environmental Engineering*
More specific subject area	*Sewage sludge combustion gaseous products*
Type of data	*Figure, Table*
How data was acquired	*The species and concentration of the main gaseous products during sludge combustion were obtained by using FTIR (DX-4000, Gasmet Technologies, Finland)*
Data format	*Raw, analyzed*
Experimental factors	– *Sewage sludge was collected from a municipal wastewater treatment plant located in the suburbs of Guangzhou, China* – *0.5 g dried sludge was used with the combustion temperature of 900 °C*, *O*_*2*_*of 3% (v/v), CO*_*2*_*of 25% (v/v), N*_*2*_*as an equilibrium gas, total gas flow rate of* 18 L/min*. Schematic of the experiment apparatus is shown as*Fig. 1 – *The species and concentration of the main gaseous products during sludge combustion process instantaneously yields are given in*Table 1
Experimental features	*Species and concentration of the main gaseous products during sludge combustion*
Data source location	*Guangzhou, China, 23°7′17″N 113°21′37″E*
Data accessibility	*Data are accessible with the article*
Related research article	*P. Fang, Z.J. Tang, X. Xiao, J.H. Huang, X.B. Chen, P.Y. Zhong, Z.X. Tang, C.P. Cen, Using sewage sludge as a flue gas denitration agent for the cement industry: Factor assessment and feasibility, J. Clean. Prod., 224, 2019, 292–303*[*1*]*.*

**Table:** 

**Value of the data** • The data provided here is important for sewage sludge disposal and cement kilns which co-dispose sewage sludge. • The data provides detail information of the species and concentration levels of the main gaseous products during sludge combustion under typical cement kiln atmosphere condition. • The data presents evidence that sewage sludge can be used as a deNOx agent in cement industry. • The data will be helpful to be referenced and compared with other researches and for future studies on the sewage sludge coordinated disposal in cement kiln.

## 1 Data

Wastewater treatment plant generate large amount of sewage sludge. Typical sewage sludge options include landfilling, incineration and enrichment of soils [2], [3], [4], [5]. The Chinese cement industry output is account for more than half of the cement output worldwide [6], thus the large quantities of NO_X_ in the flue gas discharging from cement industry deserve special attention. In this dataset, the species and concentrations of gaseous products during sludge combustion were obtained to evaluate the feasibility of using sewage sludge as a deNO_X_ agent in cement industry.

**Fig. 1:**
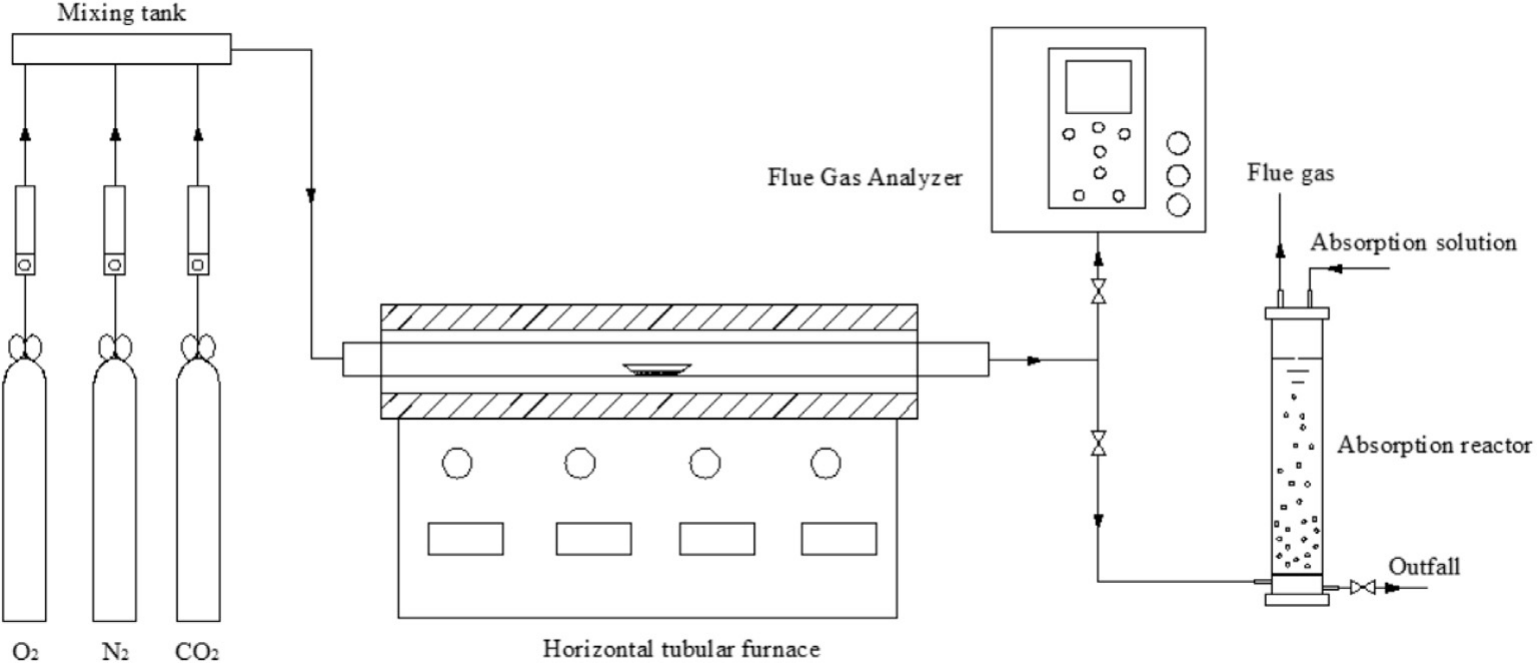
Schematic of the horizontal tubular furnace reactor system.

This dataset contains 1 figure and 1 table. Fig. 1 presents the schematic of the horizontal tubular furnace reactor system, which simulated the typical cement kiln reaction atmosphere conditions. Table 1 list the instantaneous concentrations of the main gaseous products during sludge combustion respectively.

**Table 1:** The instantaneous concentrations of the main gaseous products during sludge combustion.

Reaction time(s)	CO (vol%)	NO (ppm)	NO_2_ (ppm)	N_2_O (ppm)	NH_3_ (ppm)	CH_4_ (ppm)	HCN (ppm)
5	0.42	525.01	14.49	17.41		1086.87	388.31
10	1.89	1230.04		26.76	3.62	8168.98	1007.6
15	1.08	931.49	16.75	46.78	11.55	4408.18	1444.9
20	0.41	296.70		30.98	26.6	634.92	520.67
25	0.17	109.91		21.41	67.99	167.7	258.39
30	0.08	35.37		20.57	44.6	39.81	94.9
35	0.05	11.99		8.5	31.66	8.23	43.86
40	0.04	6.30		7.47	29.79	4.27	22.79
45	0.03	7.92		6.61	27.48	1.86	14.34
50	0.03	11.09		5.64	23.24	0.97	13.94
55	0.03	7.97		8.44	26.35	0.31	7.08
60	0.02	8.39		6.57	23.11		6.16
65	0.02	12.29		3.15	24.26		4.11
70	0.02	9.53		3.16	22.45		6.9
75	0.02	4.47		2.6	21.16		6.33
80	0.02	3.21		1.94	19.92		5.2
85	0.02	0.99		1.61	18.58		2.49
90	0.01	0.91		1.36	17.81		0.89
95	0.02	1.59		1.12	18.59		
100	0.02	2.23		0.9	16.74		
105	0.02	4.54		0.62	16.32		
110	0.02	0.17		1.93	17.59		
115	0.01	4.34		2.17	14.94		
120	0.01	0		1.84	14.68		
125	0.01	3.72		2.06	16.26		
130	0.01	3.11		1.92	14.82		
135	0.01	0		1.56	14.89		
140	0.01	2.88		0.79	14.53		
145	0.01	3.03			14.65		
150	0.01				11.82		
155	0.01				12.77		
160	0.01				10.48		
165	0.01				12.09		
170	0.01				11.91		
175	0.01				8.79		
180	0.01				10.8		
185	0.01				8.2		
190	0.01				10.37		

## 2 Experimental design, materials, and methods

### 2.1 Collection and preparation of sample

The dewatered sewage sludge sample was collected from a municipal wastewater treatment plant located in Guangzhou, China. The preparation and characterization are shown in the research article Fang et al., 2019 [1].

### 2.2 Experimental procedure

Experiments were conducted in a custom-built horizontal tubular furnace reactor (Fig. 1), which was different from the vertical fluidized-bed reactor used in Fang et al., 2019 [1]. The reactor is composed of a simulated gas unit, a horizontal tubular furnace, a flue gas sampling and online detection unit, and an absorption unit. N_2_, O_2_ and CO_2_ were obtained from cylinders and metered by mass flow controllers (Beijing seven-star electronics Co., Ltd., China). N_2_ was selected as the equilibrium gas, O_2_ of 3% (v/v), and CO_2_ of 25% (v/v) were used to compose the reaction gas to simulate the typical cement kiln atmosphere [7]. The total simulated gas flow rate was 18 L/min. After mixed in the mixing tank, the simulated gas went through the horizontal tubular furnace (14 cm in diameter and 120 cm in length). 0.5 g dried sludge in the quartz boat was pushed into the 900 °C flat-temperature zone after the gas mixture introduced into the furnace for 5 min, instead of using a screw feeder in Fang et al., 2019 [1]. The species and instantaneous concentration of the gaseous products during the sludge combustion in the outlet of the furnace were measured by using a portable FTIR analyzer (DX-4000 Gasmet Technologies, Finland). All the data were collected by computer automatically every 5 s, which tabulated in Table 1.

## References

[1] Fang P., Tang Z.J., Xiao X., Huang J.H., Chen X.B., Zhong P.Y., Tang Z.X., Cen C.P. (2019). Using sewage sludge as a flue gas denitration agent for the cement industry: factor assessment and feasibility. J. Clean. Prod..

[2] Yang K., Zhu Y., Shan R., Shao Y., Tian C. (2017). Heavy metals in sludge during anaerobic sanitary landfill: speciation transformation and phytotoxicity. J. Environ. Manag..

[3] Huang Y.Y., Li H.X., Jiang Z.W., Yang X.J., Chen Q. (2018). Migration and transformation of sulfur in the municipal sewage sludge during disposal in cement kiln. Waste Manag..

[4] Donner E., Brunetti G., Zarcina B., Harris P., Tavakkoli E., Naidu R., Lombi E. (2013). Effects of chemical amendments on the lability and speciation of metals in anaerobically digested biosolids. Environ. Sci. Technol..

[5] Westerhoff P., Lee S., Yang Y., Gordon G.W., Hristovski K., Halden R.U., Herckes P. (2015). Characterization, recovery opportunities and valuation of metals in municipal sludges from U.S. wastewater treatment plant nationwide. Environ. Sci. Technol..

[6] Fu S.L., Song Q., Tang J.S., Yao Q. (2014). Effect of CaO on the selective non-catalytic reduction deNOX process: experimental and kinetic study. Chem. Eng. J..

[7] Fang P., Tang Z.J., Huang J.H., Cen C.P., Tang Z.X., Chen X.B. (2015). Using sewage sludge as a denitration agent and secondary fuel in a cement plant: a case study. Fuel Process. Technol..

